# Effect of Phosphorylation on the Structure and Emulsification Properties of Different Fish Scale Gelatins

**DOI:** 10.3390/foods11060804

**Published:** 2022-03-11

**Authors:** Mei Yang, Jian Zhang, Xin Guo, Xiaorong Deng, Shihua Kang, Xinrong Zhu, Xiaobing Guo

**Affiliations:** Food College, Shihezi University, Shihezi 832003, China; 20192111016@stu.shzu.edu.cn (M.Y.); zhangjian0411@163.com (J.Z.); guoxin24yjs@163.com (X.G.); dxr20099@163.com (X.D.); ksh20192011005@163.com (S.K.); zhxr200208@163.com (X.Z.)

**Keywords:** fish scale gelatin, phosphorylation, structure, emulsification properties

## Abstract

This study aimed to investigate the effect of phosphorylation on the structure and emulsification of *Coregonus peled*, *Esox lucius* and *Grass carp* scale gelatin. Fourier transform infrared spectroscopy (FTIR) and endogenous fluorescence spectra showed that the structures of the three fish scale gelatins changed. Additionally, the surface hydrophobicity index of the three fish scale gelatins increased by 36.72, 31.42 and 111.67, respectively, after 1 h of phosphorylation, and the surface tension decreased by 17.27, 32.58 and 18.7 mN/m, respectively. The emulsification activity index increased by 115.86, 155.22 and 45.52 m^2^/g, and the emulsification stability index increased by 98.37, 256.77 and 169.61 min, respectively. The structure of fish scale gelatin changed after phosphorylation, which resulted in the improvement of emulsification. This work will provide useful information to understand the relationship between the structure and function of gelatin.

## 1. Introduction

Gelatin is a high molecular weight polypeptide, obtained by partial hydrolysis of collagen, and it is widely used in food, medicine, photography and other technical fields, used as a thickener, stabilizer, emulsifier, foaming agent or gelling agent [1]. Gelatin has been used as an emulsifier to stabilize food emulsions, potentially reducing the risk of chemical oxidation as well as the taste and smell of oils [2]. At present, gelatin is mainly obtained from by-products of mammals, such as cowhide, beef bone and pork skin [3]. However, religious dietary restrictions of about 23% of consumers around the world, particularly Muslim and Jewish, reject mammalian gelatin that does not meet the requirements of halal food [1]. Furthermore, mammalian gelatin also has some food safety issues. For example, contamination by animal pathogenic carriers leads to bovine spongiform encephalopathy, infectious spongiform encephalopathy, foot-and-mouth disease and hand-foot-mouth disease [4]. Gelatin from aquatic sources has been confirmed to have no risk of viral infection [3]. There is an increasing demand for alternative sources of gelatin because of social, cultural and health considerations. Fish gelatin can be obtained from fish by-products, such as skin, bones and scales. From an economic point of view, it is beneficial to the fish industry to use fish by-products to obtain gelatin [5]. In addition, fish gelatin has similar multifunctional properties (foaming, emulsification, gelation, etc.) to mammalian gelatin. Therefore, fish gelatin can be used as an ideal substitute for mammalian gelatin [1].

Fish gelatin is different from mammalian gelatin, which has a weaker emulsifying ability. Some scholars aimed to enhance the functional properties of fish gelatin via modifications, such as the incorporation of electrolytes or non-electrolytes and mechanical treatment (high pressure, ultrasonic, ultraviolet, drying and radiation) [6]. However, the formation of complex coacervates and physical treatments were highly unstable under different conditions. Chemical reagents also can enhance the functional properties of fish gelatin by cross-linking fish gelatin in ways such as phosphorylation modification, aldehyde cross-linking agent and so on. Nevertheless, there are food safety concerns because the use of aldehyde cross-linking agents might lead to genotoxic effects in the food system. Enzymatic modification like transglutaminase is widely used [7], but its application is limited by the high cost of the enzyme [6]. The introduction of phosphate groups into the protein chain can promote the hydrophobic interaction of the protein between the oil droplet interface and the surface, which leads to the improvement of emulsion stability [8]. Furthermore, studies on the digestibility of phosphorylated proteins in vivo and in vitro show that phosphorylation did not significantly reduce the nutritional value of protein. A variety of chemical substances, such as phosphorus trichloride (POCl_3_), sodium trimetaphosphate (STMP), sodium tripolyphosphate (STP) and phosphokinase, have been used to achieve phosphorylation of food proteins, but only POCl_3_ and STMP proved to be economical and practical reagents for large-scale applications [9].

Commercial cold water fish skin gelatin and bovine bone gelatin have different emulsion stability, and the percentage of β-antiparallel in the secondary structure affects the functional properties according to T. Zhang et al. [4]. T. Zhang et al. [2] also studied the effects of different extraction methods on the stability of fish skin gelatin emulsions and the stability mechanism of fish skin gelatin stabilized emulsions, involving a “protein secondary structure—molecular interaction—emulsion droplet structure—emulsion stability” route. In a previous study, Huang et al. [1] investigated the effects of phosphorylation times on the gel and emulsifying properties of fish gelatin but the authors did not study the effect of phosphorylation on the chemical structure of fish gelatin, including the primary, secondary and tertiary levels of proteins structure. However, there is still a lack of systematic study on phosphorylation modification of the functional and structural properties of fish gelatin, especially for its modification mechanism.

In view of this, the *Coregonus peled*, *Esox lucius* and *Grass carp* scales were selected for this study. The fish scale gelatin was extracted by acid method and phosphorylate by STMP. To study the changes in different fish gelatin physical and chemical properties, sodium dodecyl sulfate-polyacrylamide gel electrophoresis (SDS-PAGE), Fourier transform infrared spectroscopy and fluorescence spectroscopy were used to analyze the chemical structure of fish gelatin. The effect of phosphorylation on the emulsification properties of fish scale gelatin is discussed herein.

## 2. Materials and Methods

### 2.1. Materials

The fish scales were obtained from fisheries co., ltd. in different regions of Xinjiang, China. *Coregonus peled* scales (CS) from Sailim Lake, *Esox lucius* scales (ES) from the Altay Fuhai area and *Grass carp* scales (GS) from Bosten Lake were transported to the laboratory by a refrigerated truck. Sunflower seed oil was purchased from local supermarkets, and chemical reagents, such as sodium pyrophosphate and hydrochloric acid, were purchased from a reagent company (Macklin Reagent Co., Ltd., Shanghai, China).

### 2.2. Extraction of Fish Scale Gelatin

The fish scales were pretreated with hydrochloric acid and extracted with water according to the method of Sha et al. [10] with some modifications. The scales were mixed with distilled water at a ratio of 1:15 and washed 1–2 times. The naturally dried fish scales were powdered using a high-speed universal grinder and stored in a desiccator. According to the material-to-liquid ratio of 1:15, the fish scales were decalcified in 0.5 mol/L HCl solution for 1 h, mechanically stirred and decalcified twice. After the decalcification was completed, the fish scales were washed and centrifuged with clean water until the pH value of the supernatant was close to distilled water. After centrifugation again, the supernatant was discarded. Distilled water was added at a ratio of 1:3, fish scales to deionized water, and the pH of the mixture was adjusted to 5.5 with 1 mol/L NaOH. The scales were incubated at 80 °C for 2 h. The scale residue was removed by centrifugation, and the filtrate was passed through a 400 mesh filter cloth and lyophilized for use.

### 2.3. Analysis of the Extraction Rate of Fish Scale Gelatin

The gelatin was extracted according to Section 2.2 and the gelatin extraction rate was calculated according to the following formula.

(1) $$E\left(\%\right)=\frac{S\times m}{M}$$

where *E* is the extraction rate of fish scale gelatin (%); *S* is the solid content of fish scale gelatin extract (%); *m* is the mass of gelatin extract (g) and *M* is the mass of dried fish scale (g).

### 2.4. The Chemical Composition of Fish Scale Gelatin

Moisture, lipid, ash and protein contents were calculated by standard methods (AOAC, 2000). According to the method of Sila et al. [3], the nitrogen content of fish scale gelatin was measured by the Kjeldahl method, and the protein conversion factor was 5.55.

### 2.5. Phosphorylation Modification of Fish Scale Gelatin

The gelatin was modified by phosphorylation according to the method of Huang et al. [1] with slight modifications. Dry fish gelatin was dissolved in distilled water at 50 °C to prepare a fish gelatin solution (6.67%, *w*/*v*). STMP was added to the fish gelatin solution at a ratio of 1:20 (*w*/*w*). NaOH solution (1 mol/L) was used to adjust the pH of the mixture to 9.0. The phosphorylation reaction was conducted at 50 °C for 0, 0.5, 1, 2 or 3 h, and subsequently, the solution was cooled to room temperature under tap water to stop the reaction. STMP was converted to Na_2_HPO_4_ and reacted with CaCl_2_ to form insoluble calcium salt at a high temperature. In this study, CaCl_2_ was added to remove excessive STMP, in terms of the molar ratio of STMP: CaCl_2_ (1:2). To remove insoluble salts, the solution was heated at 90 °C for 3 min and then centrifuged at 4192.5 g for 15 min at room temperature (23 ± 1 °C). The liquid supernatants were denoted as 0, 0.5, 1, 2 and 3 h, respectively, in terms of the phosphorylation duration. The unmodified fish gelatin was named as control. All solutions were lyophilized.

### 2.6. Solubility

The fish gelatin solution (2 mg/mL) was centrifuged at 2795× *g* for 15 min at 4 °C and then the concentration of fish gelatin in the supernatant was determined. The solubility was expressed as the ratio of the concentration of the supernatant to the original fish gelatin. The calculation is as follows:

(2) $$S\left(\%\right)=\frac{c}{C}\times 100\%$$

where *S* is the solubility; *c* is the concentration of the supernatant; and *C* is the original concentration.

### 2.7. Phosphorus Content of Gelatin

According to the method of Huang et al. [1], inductively coupled plasma mass spectrometry (ICPMS 8800, Agilent Instruments, Palo Alto, CA, USA) was used to determine the content of phosphorus in unmodified fish gelatin and phosphorylated fish gelatin. First, 69% nitric acid (1 mL) was added to 0.25 g dried fish gelatin and heated by microwave until it was completely dissolved. The mixture was transferred to a 25 mL volumetric flask, and an appropriate amount of 2% nitric acid was added to achieve a constant volume of 25 mL.

### 2.8. Zeta Potential

According to the method of Kaewruang et al. [11], gelatin samples were dissolved in distilled water at a concentration of 0.05 mg/mL. Zeta potential analysis was performed using a nanoparticle zeta potential analyzer (NanoPIUS-3, Mike Instruments, New York, NY, USA) at room temperature. Before analysis, the samples were adjusted to pH 7 with 1 mol/L NaOH.

### 2.9. Amino Acid Composition

The gelatin was dissolved in ultrapure water, and 6 mol/L HCl containing 0.1% phenol was added to the gelatin solution (1 mg/mL). The sample was further hydrolyzed in a digestion tube at 110 °C for 24 h. After the sample was hydrolyzed, it was vacuum-dried again, dissolved in sodium citrate buffer and injected into the automatic amino acid analyzer (L-8900, Hitachi, Tokyo, Japan).

### 2.10. Electrophoresis Research (SDS-PAGE)

According to the method of Díaz-Calderón et al. [12], the molecular weight distribution of fish scale gelatin was analyzed by SDS-PAGE. The gelatin solution (2 mg/mL) was dissolved in 2× loading buffer at room temperature (25 ± 1 °C). One group of samples was reduced and the other group was non-reduced. The samples were denatured at 100 °C for 5 min. The samples were analyzed by 5% concentration gel and 7.5% separation gel in the electrophoresis device. The protein band was stained with Coomassie Brilliant Blue R-250 for 30 min.

### 2.11. The Chemical Structure of Fish Scale Gelatin

#### 2.11.1. FTIR Spectrum Analysis

In a Fourier transform infrared spectrometer (Bruker Vertex 70V, Shanghai, China), the FTIR spectrum of the prepared gelatin was recorded between 400 and 4000 cm^−1^. The transmission spectrum of the sample was recorded using KBr particles containing 0.1% of the sample.

#### 2.11.2. Determination of Endogenous Fluorescence Spectroscopy

According to the method of Xu et al. [13], the spatial conformation of fish scale gelatin was measured at 25 °C using a fluorescence spectrometer. Fish scale gelatin was dissolved in a phosphate buffer solution with a pH of about 7.0 and a concentration of 10 mmol/L, and a test sample solution was prepared with a fish scale gelatin concentration of 0.2 mg/mL. The test parameters of the fluorescence spectrometer were selected as follows: excitation wavelength of 295 nm, slit width of 10 nm and fluorescence emission spectra in the range of 265–320 nm were collected. The blank control was a phosphate buffer solution without fish scale gelatin.

#### 2.11.3. Determination of Surface Hydrophobicity Index (H_0_)

According to the method of Xu et al. [13], the surface hydrophobicity index of fish scale gelatin was measured with 1-anilino-8-tea sulfonate ammonium salt (ANS ammonium salt) as a fluorescent probe. Fish scale gelatin was dissolved in pH 7.0 phosphate buffer solution with different concentrations of 0.02, 0.05, 0.1, 0.2, 0.5 and 1 mg/mL. ANS was prepared at a concentration of 8 mM, using the same buffer solution, and it was stored in the dark for further use. Before detection, 20 μL of ANS solution were added to 4 mL fish scale gelatin solution, and the fluorescence intensity of the mixed solution was quickly measured by fluorescence spectrophotometer. Parameter settings: excitation wavelength of 390 nm, emission wavelength of 470 nm, slit width of 10 nm, sensitivity of 2 and test temperature of 25 °C. The relative fluorescence intensity value of the sample solution was the difference between the fluorescence intensity value of the fish scale gelatin solution with ANS added and the fluorescence intensity value without ANS. The relative fluorescence intensity value of the fish scale gelatin solution was plotted against the concentration of the fish scale gelatin solution. The initial slope of the curve was the surface hydrophobicity index (H_0_) of the gelatin sample to be tested.

### 2.12. Determination of Surface Tension

According to the method of Chen et al. [14], the surface tension of 0.1% fish gelatin solution was measured by an automated surface tensiometer (A60/A80 series, KINO Industry Co., Ltd., Boston, MA, USA). Before the measurement, the platinum ring was burned and hung in the surface tensiometer. The gelatin solution was then poured into a petri dish and placed into the surface tensiometer. The deck was adjusted to the right position and the test was started. The surface tension was recorded at the end of the test.

### 2.13. Determination of Emulsification of Fish Scale Gelatin

The emulsification of fish gelatin was determined according to the method of Xu et al. [15] with slight modifications. First, 0.1% gelatin solution was prepared and the pH of the mixture was adjusted to 9 with sodium hydroxide or hydrochloric acid (0.1 or 1 mol/L). According to previous work, at pH 9, the gel strength and emulsifying activity of phosphorylated protein were much higher than those of native protein fish oil-loaded [16], what is more, gelatin-stabilized emulsions at pH 9 exhibited better droplet stability than those at other pH values [4]. At alkaline pH, the proteins became deprotonated and had a dominant negative charge. Thus, this might help the repulsion between droplets. Sunflower oil was added to the gelatin solution in a ratio of 1:3. The mixture was homogenized at 12,000 rpm for 1 min by a high-shear emulsifying disperser. Then, 25 μL of the emulsion was transferred to a test tube. At the end of emulsification or 10 min after emulsification, 10 mL of 0.1% sodium dodecyl sulfate (SDS) solution was added for dilution. The absorbance of the diluted emulsion at 500 nm was measured by a spectrophotometer. Emulsification activity index (EAI) and emulsification stability index (ESI) are calculated by the following equations:

(3) $$EAI\;\left(m^{2}/g\right)=\frac{2\times 2.303\times A_{0}\times DF}{c\times \emptyset \times 10,000}$$

(4) $$ESI\;\left(min\right)=\frac{A_{0}}{A_{0}-A_{10}}\times 10$$

where *A_0_* is the absorbance of the emulsion after emulsification; *DF* is the dilution factor; *c* is the weight of protein per volume (g/mL); ∅ is the volume fraction of oil in the emulsion and *A_10_* is the absorbance of the emulsion diluted 10 min after emulsification.

### 2.14. Determination of Interface Adsorbed Protein

The interface adsorbed protein of fish gelatin was determined according to the method of Taha et al. [17] with some modifications. The emulsion was prepared according to the method in Section 2.13, and the protein content of the emulsion was determined by the biuret method [18]. The emulsion was centrifuged at 11,180× *g* for 0.5 h, and the lower clear liquid was collected with a syringe to determine the protein content. The calculation of the interface adsorbed protein is as follows:

(5) $$AP\left(\%\right)=\frac{w_{t}-w_{s}}{w_{t}}\times 100$$

where *AP* is the interface adsorbed protein; *W_t_* is the protein content in the original emulsion (mg) and *W_s_* is the protein content in the filtrate obtained after centrifugation.

### 2.15. Statistical Analysis

All the experiments were conducted three times and sampling is random. Statistical analysis between and within groups was done based on one-way analysis of variance (ANOVA) with Duncan’s test (*p* < 0.05) using SPSS 18.0 software (SPSS Inc., Chicago, IL, USA). The results were described as the mean values ± standard deviation.

## 3. Results and Discussion

### 3.1. The Yield and Basic Composition of Fish Gelatin

Considering the structural composition of fish scales and the economic benefits of actual production, this experiment used hydrochloric acid for decalcification. Table 1 shows the extraction yield of three kinds of fish scale gelatin following: GS > ES > CS. The trend in extraction yield may be linked to many factors, such as season, age, breed and process type [3]. Sha et al. [19] found the best pH value for fish gelatin preparation was 5.5, using yield and gel strength as evaluating indexes. Appropriate acidic conditions can destroy the non-covalent bonds in collagen, so that the collagen subunits can be easily dissolved in the later hot water extraction, thereby obtaining gelatin with higher yield and gel strength. Gelatin is mainly composed of protein, moisture, ash and fat. The protein content of the three fish scale gelatins was all above 90%. The fat content was lower than 1%, and the moisture content was lower than 6%. These results indicate that gelatin extracted in this experiment is considered edible [20]. The ash content was less than 1.3%, which is below the recommended maximum ash content of gelatin (2.6%). This indicates that the inorganic matter on the surface of the fish scales, with hydroxyapatite as the main component, was effectively removed during the decalcification process, and the quality was improved [21].

### 3.2. Solubility

As shown in Table 2, the CS gelatin solubility increased 12.39% and 12.48% after being phosphorylated for 2 h and 3 h, respectively. The ES gelatin and GS gelatin solubility increased 5.81% and 4.96% after being phosphorylated for 1 h, respectively. The subunit contents in gelatin and the hydrophobicity of gelatin itself may affect solubility. Lower subunit content and more exposed hydrophilic groups in gelatin are associated with better solubility [22]. The results of CS gelatin are inconsistent with those of ES gelatin and GS gelatin. The CS gelatin solubility decreased after being phosphorylated for 0, 0.5 h and 3 h. The ES gelatin solubility decreased after being phosphorylated for 0.5, 2 h and 3 h. The GS gelatin solubility decreased after being phosphorylated for 0, 0.5, 2 h and 3 h. The hydrophile balance affects the solubility of the protein, and the solubility depends on the amino acid composition, especially on the surface of the protein. Upon phosphorylation treatment, protein side chain groups selectively induce a large number of phosphate groups. As the negative charge introduced by the phosphate groups on the surface of the protein molecule increases, the hydration of the protein is enhanced, thereby increasing its water solubility [23,24,25]. Phosphorylation treatment can cause protein denaturation, thereby breaking the intramolecular hydrogen bonds, destroying the spatial structure of the protein and turning it into a long chain of amino acids. In this way, it was easy to form intermolecular hydrogen bonds between chains, leading to aggregate clusters, which are not easily dissolved. Therefore, the solubility of fish gelatin is reduced at the phosphorylation time mentioned above.

### 3.3. Element Content and Zeta Potential

Table 3 shows that after phosphorylation, the P content of gelatins of the three fish scales increased. In this experiment, STMP was reacted with CaCl_2_ at high a temperature, and excess STMP was removed by centrifugation. Therefore, Ca^2+^ and Na^+^, which could affect the gelation ability of fish gelatin, were artificially introduced, and their content was monitored. The control sample was not treated; thus, it had lower levels of calcium and sodium. The reason for the fluctuation of phosphorus content may be related to the raw material fish scale. Factors such as season, age, variety and type of process may all affect the properties of gelatin. Another reason is that high temperature could convert STMP into Na_2_HPO_4_, which could react with CaCl_2_ to form less-soluble calcium salt. In this study, excessive STMP was removed by adding CaCl_2_ according to the molar ratio of STMP: CaCl_2_ (1:2). It is possible that phosphates react with calcium salts during this process, forming precipitates that are removed together.

Table 3 also shows that the zeta potential of the three fish scale gelatins was the highest at 0.5 h, compared to the control. Because phosphate attachment increased the net negative charge, the zeta potential of fish glue increased with the addition of phosphate. The charge amount and charge properties of gelatin are regulated by amino acids on the α-chain. Arg is rich in free amino groups and has a positively charged surface after protonation, which is favorable for binding to negatively charged phosphates. The measurement was made to monitor the charge of modified protein and Calcium was used for the removal of excessive phosphate used, so the negative charge was not dominant. The zeta potential can affect the stability of colloidal dispersions and shows the degree of repulsion between similarly charged particles [26]. In the emulsion system, by introducing negatively charged phosphate groups, the droplets gain a strong electrostatic repulsive force, which improves the surface charge intensity of the droplets. This leads to better spatial stability and prevents aggregation and coalescence between the droplets [23,27,28]. For samples with reduced zeta potential, this may be attributed to the formation of aggregates, because the decrease in surface charge may be related to the exposure of hydrophobic non-polar residues through the expansion of the tertiary structure. The inconsistency between the results of potential and solubility should be related to the introduction of Ca^2+^ and Na^+^.

### 3.4. Amino Acid Composition

Chemical composition, amino acid composition and molecular weight distribution are the main factors affecting the properties of gelatin. Amino acids are relevant to the stability of the triple helix of collagen and gel structure through hydrogen bonding [27]. The three main amino acids of gelatin are glycine, alanine and proline. The high glycine content has an important effect on the spatial structure. In fact, the triple-stranded helical structure of collagen has glycine at every third position in the sequence. Furthermore, collagen contains high content of proline and hydroxyproline. Table 4, Table 5 and Table 6 reveals that the three fish scale gelatins had low tyrosine and histidine content, and none of them contained cysteine and tryptophan, because there are usually not present in gelatin. These results are consistent with the amino acid composition of gelatin extracted from European eel skin [3].

The total amino acid contents of CS gelatin decreased at phosphorylation for 2 h, and that of GS gelatin decreased at phosphorylation for 0.5 h. However, after being phosphorylated for 1 h, the amino acid content of ES gelatin decreased with increasing treatment time. The reason was that the amino acids of the protein side chain groups react with the phosphate groups through phosphorylation and generate phosphorous amino acid residues [23]. According to the previous report, the mechanism of modification of food protein by phosphate is that the protein side chain groups, including -OH groups of Ser, Thr and Tyr, ε-NH_2_ of Lys, the 1 and 3 nitrogen atoms of the His imidazole ring and the nitrogen atom of the Arg guanidine group, were selectively induced by a large number of phosphate groups [24]. The hydrophilicity/hydrophobicity balance influences the protein solubility, which depends on the amino acid composition, particularly at the protein surface [8]. Under phosphorylated treatment, protein side chain groups selectively induced a large number of phosphate groups. With the increasing negative charges introduced by phosphate groups along the protein molecular surface, the hydration of protein will be enhanced and lead to the improvement in water solubility [8,24]. The phosphorylation of the lysine site in gelatin hinders the hydrolysis of lysine residues in gelatin and reduces the content of lysine, so the content of amino acids is reduced [28]. The three kinds of fish scale gelatins had different reduction rules of amino acids, which was due to different degrees of phosphorylation reaction. This trend can be inferred from the element contents in Table 3. Mutilangi et al. [29] found that the stability of the emulsion was positively correlated with the molecular weight of the polypeptide or the content of the hydrophobic polypeptide. Alanine, valine, leucine, isoleucine, proline, hydroxyproline and methionine are hydrophobic amino acids. CS gelatin, ES gelatin and GS gelatin had the highest hydrophobic amino acid content after 1, 0.5 and 2 h phosphorylation, which was 28.29, 27.94 and 28.72 g/100 g, respectively. ES gelatin had a higher hydrophobic amino acid content after 1 h phosphorylation, at 24.42 g/100 g; GS gelatin had higher hydrophobic amino acid contents after 3 h and 1 h phosphorylation, at 27.92 and 25.55 g/100 g, respectively.

### 3.5. SDS-PAGE

**Figure 1:**
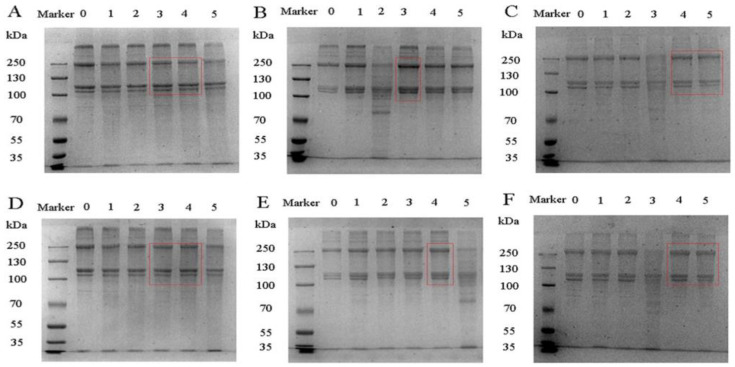
SDS map of fish gelatin. (**A**) (0–5): CS gelatin reduced, CS gelatin reduced at phosphorylation 0, 0.5, 1, 2 and 3 h, respectively; (**B**) (0–5): ES gelatin reduced, ES gelatin reduced at phosphorylation 0, 0.5, 1, 2 and 3 h, respectively; (**C**) (0–5): GS reduced, GS gelatin reduced at phosphorylation 0, 0.5, 1, 2 and 3 h, respectively; (**D**) (0–5): CS gelatin non-reduced, CS gelatin non-reduced at phosphorylation 0, 0.5, 1, 2 and 3 h, respectively; (**E**) (0–5): ES gelatin non-reduced, ES gelatin non-reduced at phosphorylation 0, 3, 2, 1 and 0.5 h, respectively and (**F**) (0–5): GS gelatin non-reduced, GS gelatin non-reduced at phosphorylation 0, 0.5, 1, 2 and 3 h, respectively.

SDS-PAGE was used to detect the subunit composition and relative molecular mass distribution of the gelatin samples. The electrophoresis results of gelatin extracted from CS, ES and GS are shown in Figure 1. During the thermal extraction process, the triple helix structure in collagen is transformed into random coils. The α-peptide chain is released and mainly retained in the gelatin in the form of α, β and γ subunit components. As shown in Figure 1, the molecular mass distributions of the three fish scale gelatins were very similar, including α1, α2 and β chains, as well as unclear small molecule fragments. The molecular weight of the α1 and α2 chains was between 100–130 kDa, and the molecular weight of the β chain was about 250 kDa. Some high molecular weight protein bands were also detected above the β chain. They may be the γ component in collagen and some high molecular protein components. Comparing the reduced and non-reduced gelatin electrophoresis bands, the protein profiles were consistent, indicating that the three fish scale gelatin structures did not contain disulfide bonds and had high purity. CS gelatin phosphorylated 1 h and 2 h, ES gelatin phosphorylated 1 h and GS phosphorylated 2 h and 3 h had darker bands, indicating that the content of α and β chains was higher. Limited hydrolysis occurs during gelatin phosphorylation, cutting off the non-helical regions at both ends. Increased α chain strength after phosphorylation correlates with solubility. The better the solubility, the greater the α chain strength. Differences in the structure of the gelatin samples led to different results. The higher the content of α and β chains, the better the stability of the emulsion, which was due to the stronger hydrophobic interaction between the emulsion and the oil [30].

### 3.6. Chemical Structure of Fish Gelatin

#### 3.6.1. Fourier Transform Infrared Spectroscopy

**Figure 2:**
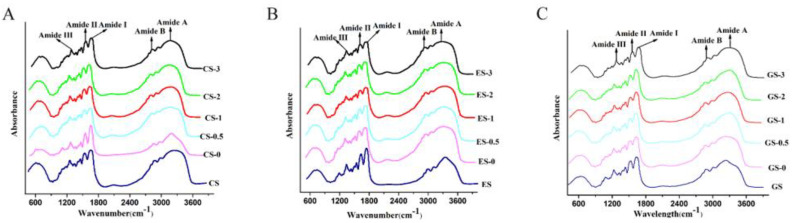
ATR-FTIR spectra of fish gelatin within the wavelength range of 4000–400 cm^−1^. (**A**) CS, CS-0, CS-0.5, CS-1, CS-2, CS-3: CS gelatin, CS gelatin phosphorylated by STMP for 0, 0.5, 1, 2 and 3 h, respectively. (**B**) ES, ES-0, ES-0.5, ES-1, ES-2, ES-3: ES gelatin, ES scale gelatin phosphorylated by STMP for 0, 0.5, 1, 2 and 3 h, respectively. (**C**) GS, GS-0, GS-0.5, GS-1, GS-2, GS-3: GS gelatin, GS gelatin phosphorylated by STMP for 0, 0.5, 1, 2 and 3 h, respectively.

From Figure 2, the FTIR absorption spectra of the three fish scale gelatins in the range of 4000 to 400 cm^−1^ showed similar spectral shapes and five regions (amide A, amide B, amide I, amide II and amide III). The vibration mode of amide I (1700–1600 cm^−1^) is mainly a C=O tensile vibration, coupled with the C-N stretching. The absorption peak at amide I is characteristic of the gelatin curl structure. Amide II (1570–1335 cm^−1^) is produced by the bending vibration of the N-H group and the tensile vibration of the C-N group. Amide III (1300–1000 cm^−1^) represents the combined peak between the C-N stretching vibration and N-H deformation of the amide bond, and the absorption is caused by the swing vibration of the CH_2_ group of the glycine main chain and the proline side chain. The amide A band (3600–3200 cm^−1^) is caused by the stretching vibration of the N-H group. The amide band associated with the N-H stretching vibration indicates the presence of hydrogen bonds. CS gelatin, ES gelatin and GS gelatin amide A bands appeared at 3337.36, 3322.03 and 3309.53 cm^−1^ but redshifted to 3385.26, 3368.69 and 3395.89 cm^−1^ after phosphorylation modification for 1 h, respectively, suggesting the involvement of an N-H group of gelatin in interaction with phosphate via ionic interaction [11]. This indicates that the secondary structure had changed. Amide B (3100–3000 cm^−1^) is related to the asymmetric tensile vibration of =C-H and -NH^3+^ [5].

The secondary structure of gelatin was analyzed, based on the area of 1700–1600 cm^−1^: peak center 1610–1642 cm^−1^ (β-sheet), peak center 1642–1650 cm^−1^ (random coil), peak center 1650–1660 cm^−1^ (α helix), peak center 1660–1680 cm^−1^ (β turn) and peak center 1680–1700 cm^−1^ (β antiparallel) [4]. The secondary structure content (β-sheet > β-turn > random coil > α-helix) of the three fish scale gelatin samples was the same as commercial fish gelatin of tilapia skin (160 Bloom) [2]. As shown in Table 7, the β-sheet content in the secondary structure of CS gelatin increased after phosphorylation, but the α-helix and β-turn content decreased. The β-sheet became dominant and was higher than α-helix. That is because phosphorylation of gelatin mainly leads to α-helical structure and β-turn structure transforms to β-sheet structure [31]. It has been reported that the β-sheet structures are relatively stable, whereas the α-helix, β-turn and random coil structures are relatively flexible and open [32]. It was reported that β-sheet played a crucial role in forming with interactions of adjacent protein molecules, and that high β-sheet content would favor in reducing the oil–water interfacial tension. Phosphorylation resulted in the formation of disorder conformation of protein, which was helpful in facilitating protein structure transformation by maintaining the interfacial flexibility of protein at the oil–water interface [33]. However, as shown in Table 8 and Table 9, ES and GS gelatin showed completely opposite trends. This result indicates that the modification of fish gelatin with STMP changes its secondary structure.

#### 3.6.2. Endogenous Fluorescence

**Figure 3:**
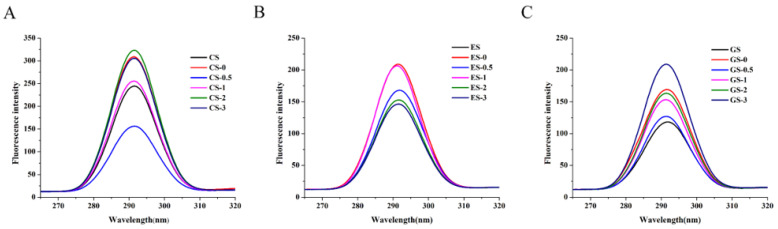
Endogenous fluorescence spectrum of fish gelatin. (**A**) CS, CS-0, CS-0.5, CS-1, CS-2, CS-3: CS gelatin, CS gelatin phosphorylated by STMP for 0, 0.5, 1, 2 and 3 h, respectively. (**B**) ES, ES-0, ES-0.5, ES-1, ES-2, ES-3: ES gelatin, ES scale gelatin phosphorylated by STMP for 0, 0.5, 1, 2 and 3 h, respectively. (**C**) GS, GS-0, GS-0.5, GS-1, GS-2, GS-3: GS gelatin, GS gelatin phosphorylated by STMP for 0, 0.5, 1, 2 and 3 h, respectively.

**Figure 4:**
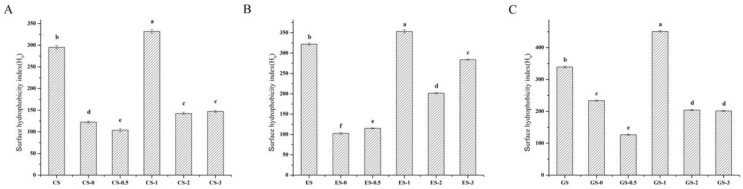
Surface hydrophobicity index of fish gelatin. (**A**) CS, CS-0, CS-0.5, CS-1, CS-2, CS-3: CS gelatin, CS gelatin phosphorylated by STMP for 0, 0.5, 1, 2, 3 h respectively. (**B**) ES, ES-0, ES-0.5, ES-1, ES-2, ES-3: ES gelatin, ES scale gelatin phosphorylated by STMP for 0, 0.5, 1, 2, 3 h respectively. (**C**) GS, GS-0, GS-0.5, GS-1, GS-2, GS-3: GS gelatin, GS gelatin phosphorylated by STMP for 0, 0.5, 1, 2, 3 h respectively. Different lowercase letters denote significant differences (*p* < 0.05).

The fluorescence intensity may be an important indicator of the degree of exposure of aromatic amino acids to water and can reflect the changes in the tertiary structure of gelatin [13,34]. The fluorescence spectrums of CS gelatin, ES gelatin and GS gelatin are shown in Figure 3A–C, respectively. The three fish scale gelatins exhibited the strongest fluorescence intensity at 291 nm. The fluorescence intensity of CS gelatin after phosphorylation for 0.5 h was lower than that of the unmodified sample. The fluorescence intensities of CS gelatin after 0, 1, 2 and 3 h phosphorylation were higher than that of the unmodified gelatin. The fluorescence intensity of CS gelatin after 2 h phosphorylation was the largest among them. The fluorescence intensities of ES gelatin after 2 and 3 h phosphorylation were lower than that of the unmodified gelatin. The fluorescence intensity of ES gelatin after 1 h phosphorylation was the largest among them. This result was coincident with latter surface hydrophobicity data (Figure 4B). The fluorescence intensity of the phosphorylated GS gelatin was higher than that of the unmodified gelatin. With increasing phosphorylation time, the fluorescence intensities after 0.5, 1, 2 and 3 h showed a gradual increase. Differences between the structure of the unmodified and modified gelatin protein were observed. The fluorescence intensity of phosphorylated gelatin was higher than that of the control because more aromatic groups were exposed on the surface of protein molecules, leading to an increase in emission fluorescence [13]. The process of phosphorylation modification may cause gelatin expansion, and gelatin will undergo denaturation and aggregation to form large gelatin molecules [23,35]. As a result, the energy transfer was weakened, so the fluorescence intensity of GS gelatin after 0.5 h phosphorylation was significantly reduced.

#### 3.6.3. Surface Hydrophobicity Index

Surface hydrophobicity measures the exposure of hydrophobic amino acids in the protein chain, otherwise, these amino acids are hidden inside the protein chain. As a fluorescent probe, ANS is non-covalently bound to the hydrophobic area of the protein surface [36]. H_0_ reflects the ability of protein to absorb at the water–air interface and oil–water interface through hydrophobic interactions. These interactions have a profound effect on the solubility, foaming, interface adsorption behavior and emulsification of the protein [37].

Figure 4 illustrates that the surface hydrophobicity index of the three kinds of fish scale gelatin significantly increased after 1 h phosphorylation. The surface hydrophobicity index of CS gelatin, ES gelatin and GS gelatin increased by 36.72, 31.42 and 111.67, respectively, compared to the control. Some scholars considered that the higher energy phosphate compounds could react with -OH or -NH_2_ groups on the side chains of proteins, resulting in higher surface hydrophobicity of fish gelatin [38]. Phosphorylation for 0, 0.5, 2 and 3 h resulted in a lower surface hydrophobicity index than that of unmodified fish scale gelatin. The increase in H_0_ is mainly related to protein unfolding or hydrolysis. This led to the discovery of hydrophobic groups buried in internal molecules, which are more exposed to the water environment. According to previous studies, the decrease in hydrophobicity may be due to (a) the destruction of the hydrophobic area by phosphorylation modification, which increases the hydrophobicity of the surface; and (b) reduction of the surface area of hydrophobic groups exposed to surrounding water through protein-protein aggregation [13,39,40]. However, the current results of enhanced surface hydrophobicity are inconsistent with the results of enhanced solubility of gelatin, which may be due to the fact that the solubility of gelatin was affected by the polymer subunits [37].

### 3.7. Surface Tension

**Figure 5:**
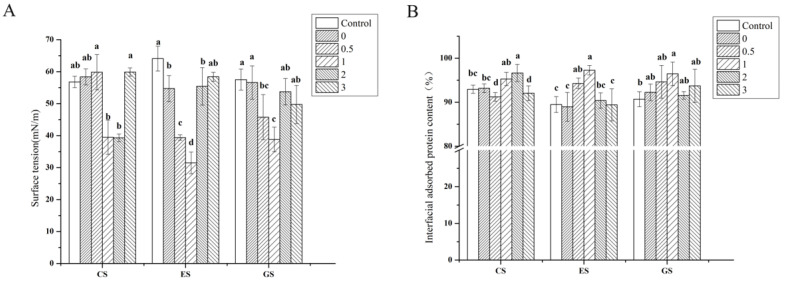
Surface tension of fish gelatin (**A**) and interface adsorption protein of fish gelatin (**B**). Control: raw fish gelatin, 0–3: phosphorylation of fish gelatin at 0, 0.5, 1, 2 and 3 h. Different lowercase letters denote significant differences (*p* < 0.05).

Gelatins are crucial for the formation of emulsions because of their amphiphilicity and film-forming ability. They can adsorb at the oil–water interface and reduce the interfacial tension, thereby improving the stability of emulsions. The higher surface hydrophobicity of gelatin is more conducive to reducing the interfacial tension and promoting its adsorption on the interface. The lower the surface tension, the more stable the emulsion [22]. As shown in Figure 5A, with the extension of phosphorylation time, the surface tension of the three fish scale gelatins first decreased and then increased. The surface tension of CS gelatin was the smallest after phosphorylation for 2 h, which was 39.29 mN/m, and its surface tension was 39.49 mN/m after 1 h phosphorylation. The surface tension of ES and GS gelatin was the smallest after 1 h phosphorylation, at 31.48 and 38.81 mN/m, respectively. This result is consistent with the surface hydrophobicity and emulsion stability results.

### 3.8. Emulsification Properties

The emulsifying activity reflects the rapid adsorption of protein at the oil–water interface during the formation of the emulsion. By inducing a negative charge on the phosphate groups in the protein chain residues, the phosphorylated fish gelatin can quickly move to the oil–water interface, and by increasing the electrostatic repulsion of the droplets, the adsorption of proteins on the oil–water interface layer and the dispersion of oil droplets are improved [23,38,41]. ESI refers to the ability of the emulsion to remain dispersed without coalescence, flocculation and emulsification.

**Figure 6:**
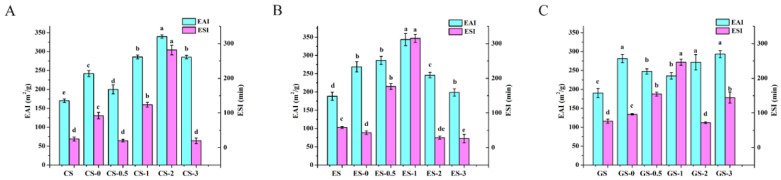
Emulsification activity index and emulsion stability index of fish gelatin. (**A**) CS, CS-0, CS-0.5, CS-1, CS-2, CS-3: CS gelatin, CS gelatin phosphorylated by STMP for 0, 0.5, 1, 2 and 3 h, respectively. (**B**) ES, ES-0, ES-0.5, ES-1, ES-2, ES-3: ES gelatin, ES scale gelatin phosphorylated by STMP for 0, 0.5, 1, 2 and 3 h, respectively. (**C**) GS, GS-0, GS-0.5, GS-1, GS-2, GS-3: GS gelatin, GS gelatin phosphorylated by STMP for 0, 0.5, 1, 2 and 3 h, respectively. Different lowercase letters denote significant differences (*p* < 0.05).

As shown in Figure 6A, with increasing phosphorylation time, the emulsification activity index and emulsification stability index of CS phosphorylated gelatin generally showed a gradually increasing trend, especially after 2 h. The emulsification activity index of CS gelatin increased by 163.65 m^2^/g, and the emulsification stability index of CS gelatin increased by 256.26 min after 2 h, compared to the control. The emulsification activity index of CS gelatin after 3 h of phosphorylation began to decrease, and the emulsification stability index of CS gelatin was lower than that of the unmodified gelatin. Taken together, the emulsification of CS gelatin phosphorylated for 2 h was the best, but the emulsification of CS gelatin after 1 h also improved. Similar to CS gelatin, as shown in Figure 6B, the emulsification activity index of ES gelatin after 1 h phosphorylation was the highest (increasing by 155.22 m^2^/g), and the emulsification stability index of ES gelatin after 1 h phosphorylation increased by 256.77 min, compared to the control. As shown in Figure 6C, the emulsification activity index of GS gelatin increased at different phosphorylation times, and there was no significant difference between the 2 and 3 h samples. The GS gelatin after 1 h phosphorylation had better emulsification. The emulsification activity index increased by 45.52 m^2^/g, and the emulsification stability index increased by 169.91 min, compared to the control. The results show the short-term modification improved the emulsification of fish gelatin, and the long-term modification was not conducive to the improvement of its emulsification stability. As shown in Figure 6, the reason why different phosphorylation times render the highest emulsifying property of different gelatins is related to the hydrophobic amino acid content of gelatins, which is explained in Section 3.4.

### 3.9. Interfacial Adsorbed Protein Content

In the process of emulsion formation, gelatin acts as a surfactant and quickly adsorbs to the oil–water interface, forming a stable interface film. The content of interface protein directly affects the thickness of the interface film; therefore, the content of interface protein is an important index to evaluate the stability of emulsion [42]. The higher the content of adsorbed protein, the more stable the emulsion. As shown in Figure 5B, the interface adsorbed protein content of CS gelatin was the largest after phosphorylation for 2 h (96.64%), and its interface adsorbed protein content was 95.28% after 1 h phosphorylation. The interface adsorbed protein content of ES gelatin and GS gelatin was the highest after 1 h phosphorylation, at 97.28% and 96.49%, respectively. This result is consistent with the emulsion stability result. The formation of soluble aggregates may be the reason for the increase in interfacial protein content in gelatin emulsions.

## 4. Conclusions

The results of this study demonstrated that phosphorylation could be used as a method to produce fish gelatin with improved emulsifying properties. Fourier transform infrared spectroscopy and endogenous fluorescence spectroscopy showed that phosphorylation changed the secondary and tertiary structure of fish scale gelatin. When phosphorylated for 1 h, the increase of surface hydrophobicity index, the decrease of surface tension and the increase of the content of interface adsorbed protein resulted in the increase of emulsification activity index and emulsion stability index. This research lays the foundation for the application of gelatin in the food industry and helps to develop dairy products with better emulsification.

## Figures and Tables

**Table 1:** Yield and basic components of fish gelatin.

	CS	ES	GS
Yield	10.18 ± 1.12 ^c^	15.7 ± 0.27 ^b^	21.78 ± 0.83 ^a^
Protein	93.61 ± 0.88 ^a^	92.94 ± 0.09 ^a^	93.25 ± 1.32 ^a^
Moisture	4.65 ± 0.31 ^a^	4.25 ± 0.08 ^a^	4.98 ± 1.15 ^a^
Ash	0.79 ± 0.04 ^b^	1.01 ± 0.02 ^a^	0.13 ± 0.01 ^c^
Fat	0.67±0.04 ^b^	0.85±0.05 ^a^	0.38±0.05 ^c^

CS: *Coregonus peled* scale gelatin, ES: *Esox lucius* scale gelatin, GS: *Grass carp* scale gelatin. For the same row, different superscripts indicate significant differences (*p* < 0.05).

**Table 2:** The effect of phosphorylation on the solubility (%) of fish gelatin.

*Coregonus peled* Scale Gelatin	Solubility	*Esox lucius* Scale Gelatin	Solubility	*Grass carp* Scale Gelatin	Solubility
CS	92.26 ± 0.74 ^b^	ES	91.74 ± 0.89 ^b^	GS	88.97 ± 7.47 ^a^
CS-0	87.47 ± 1.99 ^c^	ES-0	93.15 ± 2.81 ^ab^	GS-0	82.46 ± 8.13 ^a^
CS-0.5	87.53 ± 1.12 ^c^	ES-0.5	90.45 ± 6.38 ^b^	GS-0.5	85.35 ± 0.64 ^a^
CS-1	85.54 ± 1.00 ^c^	ES-1	98.56 ± 2.31 ^a^	GS-1	86.55 ± 4.23 ^a^
CS-2	98.31 ± 1.38 ^a^	ES-2	91.45 ± 0.84 ^b^	GS-2	82.43 ± 0.10 ^a^
CS-3	98.39 ± 0.87 ^a^	ES-3	90.86 ± 0.54 ^b^	GS-3	81.11 ± 0.61 ^a^

CS, CS-0, CS-0.5, CS-1, CS-2, CS-3: CS gelatin, CS gelatin phosphorylated by STMP for 0, 0.5, 1, 2 and 3 h, respectively. ES, ES-0, ES-0.5, ES-1, ES-2, ES-3: ES gelatin, ES scale gelatin phosphorylated by STMP for 0, 0.5, 1, 2 and 3 h, respectively. GS, GS-0, GS-0.5, GS-1, GS-2, GS-3: GS gelatin, GS gelatin phosphorylated by STMP for 0, 0.5, 1, 2 and 3 h, respectively. For the same column, different superscripts indicate significant differences (*p* < 0.05).

**Table 3:** The contents of sodium, calcium, phosphate in fish gelatin and ζ-potential values of phosphorylated fish gelatin solution.

Gelatin	P (mg/kg)	Ca (mg/kg)	Na (g/kg)	ζ-Potential (mv)
CS	294.70 ± 4.48 ^a^	203.43 ± 10.52 ^b^	5.14 ± 0.22 ^d^	0.58 ± 0.16 ^b^
CS-0	705.31 ± 5.32 ^e^	327.63 ± 3.17 ^f^	110.38 ± 8.95 ^ab^	0.11 ± 1.13 ^bc^
CS-0.5	888.31 ± 3.39 ^d^	542.32 ± 7.53 ^d^	102.70 ± 0.65 ^c^	1.87 ± 0.12 ^a^
CS-1	965.62 ± 10.77 ^b^	913.03 ± 10.71 ^c^	108.87 ± 4.48 ^c^	0.40 ± 0.06 ^b^
CS-2	435.52 ± 6.19 ^f^	963.85 ± 10.78 ^a^	112.80 ± 7.46 ^ab^	0.33 ± 0.01 ^b^
CS-3	896.61 ± 10.6 ^c^	372.15 ± 4.26 ^e^	122.46 ± 10.18 ^a^	−0.69 ± 0.16 ^c^
ES	82.75 ± 3.34 ^f^	139.40 ± 1.16 ^e^	4.08 ± 0.31 ^b^	13.44 ± 0.83 ^a^
ES-0	673.74 ± 2.37 ^e^	261.82 ± 4.11 ^a^	105.11 ± 3.60 ^a^	0.48 ± 0.14 ^cd^
ES-0.5	786.37 ± 8.66 ^a^	213.16 ± 1.21 ^b^	111.29 ± 7.30 ^a^	1.47 ± 0.55 ^b^
ES-1	711.31 ± 4.66 ^d^	199.28 ± 4.85 ^d^	106.19 ± 2.01 ^a^	0.19 ± 0.36 ^de^
ES-2	722.35 ± 9.34 ^b^	196.00 ± 1.42 ^d^	112.09 ± 5.64 ^a^	−0.43 ± 0.10 ^e^
ES-3	713.96 ± 5.92 ^c^	204.87 ± 2.03 ^c^	105.21 ± 5.28 ^a^	1.31 ± 0.46 ^bc^
GS	188.79 ± 2.53 ^f^	73.35 ± 1.48 ^d^	6.81 ± 0.50 ^d^	5.05 ± 0.32 ^a^
GS-0	971.71 ± 1.71 ^a^	200.72 ± 5.17 ^c^	100.03 ± 3.79 ^c^	−0.42 ± 0.14 ^f^
GS-0.5	655.64 ± 4.46 ^e^	664.40 ± 6.03 ^a^	103.65 ± 2.45 ^c^	2.63 ± 0.21 ^b^
GS-1	837.98 ± 7.64 ^c^	491.16 ± 9.00 ^b^	124.24 ± 9.37 ^a^	0.05 ± 0.20 ^e^
GS-2	967.91 ± 7.07 ^b^	443.21 ± 4.41 ^b^	113.59 ± 7.68 ^ab^	1.38 ± 0.15 ^c^
GS-3	705.34 ± 1.77 ^d^	611.85 ± 10.4 ^a^	126.61 ± 4.72 ^a^	0.39 ± 0.12 ^d^

CS, CS-0, CS-0.5, CS-1, CS-2, CS-3: CS gelatin, CS gelatin phosphorylated by STMP for 0, 0.5, 1, 2 and 3 h, respectively. ES, ES-0, ES-0.5, ES-1, ES-2, ES-3: ES gelatin, ES scale gelatin phosphorylated by STMP for 0, 0.5, 1, 2 and 3 h, respectively. GS, GS-0, GS-0.5, GS-1, GS-2, GS-3: GS gelatin, GS gelatin phosphorylated by STMP for 0, 0.5, 1, 2 and 3 h, respectively. For the same column, different superscripts indicate significant differences (*p* < 0.05).

**Table 4:** Amino acid composition (Amino acids g/100 g protein) of *Coregonus peled* scale gelatin.

	CS	CS-0	CS-0.5	CS-1	CS-2	CS-3
Asp	5.84	6.80	6.92	6.99	4.73	6.94
Thr	2.38	2.80	2.85	2.87	1.95	2.83
Ser	4.15	4.83	4.91	4.97	3.36	4.94
Glu	9.47	11.02	11.25	11.35	7.75	11.30
Gly	22.80	26.56	27.04	27.27	18.41	27.04
Ala	8.75	9.93	10.60	10.70	7.17	10.63
Val	1.71	1.99	2.03	2.04	1.41	1.13
Met	1.77	2.02	1.28	1.41	0.72	1.29
Ile	1.06	1.24	1.26	1.25	0.85	1.25
Leu	2.07	2.42	2.46	2.47	1.71	2.48
Tyr	0.20	0.26	0.25	0.26	0.16	0.28
Phe	1.91	2.22	2.25	2.28	1.60	2.26
Lys	3.08	3.61	3.69	3.72	2.51	3.69
His	1.20	1.36	1.38	1.40	0.94	1.39
Arg	7.80	9.15	9.33	9.42	6.32	9.34
Pro	8.83	10.02	10.36	10.42	7.67	10.45
Total	83.03	96.21	97.84	98.82	67.25	97.24

CS, CS-0, CS-0.5, CS-1, CS-2, CS-3: *Coregonus peled* scale gelatin, *Coregonus peled* scale gelatin phosphorylated by STMP for 0, 0.5, 1, 2 and 3 h respectively.

**Table 5:** Amino acid composition (Amino acids g/100 g protein) of *Esox lucius* scale gelatin.

	ES	ES-0	ES-0.5	ES-1	ES-2	ES-3
Asp	6.41	6.72	6.77	5.89	4.45	3.14
Thr	2.74	2.87	2.90	2.53	1.97	1.35
Ser	3.93	4.14	4.16	3.65	2.76	1.94
Glu	10.14	10.64	10.67	9.32	7.05	4.95
Gly	24.38	25.64	25.70	22.41	16.76	11.91
Ala	10.16	10.64	10.70	9.32	6.99	4.97
Val	1.96	1.98	2.03	1.77	1.39	0.94
Met	0.21	0.01	0.70	0.50	0.36	0.54
Ile	1.19	1.24	1.21	1.06	0.78	0.56
Leu	2.38	2.50	2.50	2.18	1.64	1.16
Tyr	0.29	0.32	0.31	0.26	0.20	0.14
Phe	2.01	2.10	2.09	1.85	1.44	0.96
Lys	3.50	3.67	3.68	3.20	2.40	1.70
His	1.64	1.72	1.74	1.51	1.14	0.82
Arg	8.59	9.04	9.06	7.88	5.84	4.19
Pro	10.33	10.82	10.81	9.59	7.76	5.05
Total	89.86	94.04	95.00	82.93	62.91	44.33

ES, ES-0, ES-0.5, ES-1, ES-2, ES-3: *Esox lucius* scale gelatin, *Esox lucius* scale gelatin phosphorylated by STMP for 0, 0.5, 1, 2 and 3 h respectively.

**Table 6:** Amino acid composition (Amino acids g/100 g protein) of *Grass carp* scale gelatin.

	GS	GS-0	GS-0.5	GS-1	GS-2	GS-3
Asp	5.14	5.20	4.89	5.48	6.01	5.94
Thr	2.36	2.39	2.24	2.49	2.77	2.73
Ser	3.44	3.52	3.34	3.73	4.09	4.03
Glu	8.65	8.83	8.27	9.17	10.18	10.08
Gly	20.94	21.16	19.88	22.25	24.53	24.29
Ala	9.45	9.52	8.87	9.92	11.04	10.93
Val	1.82	1.85	1.75	1.96	2.12	2.10
Met	0.95	0.79	0.11	0.31	1.14	0.41
Ile	1.01	1.00	0.93	1.02	1.15	1.15
Leu	2.34	2.39	2.25	2.53	2.75	2.74
Tyr	0.28	0.25	0.22	0.26	0.28	0.31
Phe	1.84	1.92	1.81	1.97	2.18	2.18
Lys	3.12	3.15	2.95	3.25	3.65	3.62
His	0.66	0.65	0.61	0.70	0.74	0.73
Arg	7.76	7.81	7.35	8.23	9.10	8.99
Pro	8.66	9.41	9.08	9.80	10.52	10.59
Total	78.41	79.84	74.55	83.1	92.24	90.81

GS, GS-0, GS-0.5, GS-1, GS-2, GS-3: *Grass carp* scale gelatin, *Grass carp* scale gelatin phosphorylated by STMP for 0, 0.5, 1, 2 and 3 h respectively.

**Table 7:** Secondary structure percentage (%) analysis of *Coregonus peled* scale gelatin by analyzing the areas of 1600–1700 cm^−^^1^ in FTIR spectra.

Sample	β-Sheet	Random Coil	α-Helix	β-Turn	β-Antiparallel
CS	45.20 ± 3.40 ^a^	13.37 ± 0.65 ^a^	12.70 ± 0.33 ^a^	24.20 ± 4.20 ^a^	4.53 ± 1.90 ^b^
CS-0	36.94 ± 3.44 ^b^	14.31 ± 0.95 ^a^	14.44 ± 0.71 ^b^	27.78 ± 5.68 ^a^	6.54 ± 0.58 ^b^
CS-0.5	43.48 ± 3.42 ^ab^	13.76 ± 0.78 ^a^	12.62 ± 0.23 ^a^	23.78 ± 4.99 ^a^	6.35 ± 0.56 ^b^
CS-1	43.36 ± 3.48 ^ab^	13.21 ± 0.55 ^a^	12.29 ± 0.21 ^a^	25.35 ± 4.73 ^a^	5.79 ± 0.50 ^ab^
CS-2	43.22 ± 3.45 ^ab^	13.69 ± 0.66 ^a^	12.77 ± 0.27 ^a^	23.91 ± 4.90 ^a^	6.41 ± 0.52 ^b^
CS-3	38.65 ± 3.33 ^ab^	13.04 ± 0.73 ^a^	12.87 ± 0.55 ^a^	28.67 ± 5.18 ^a^	6.77 ± 0.58 ^b^

CS, CS-0, CS-0.5, CS-1, CS-2, CS-3: *Coregonus peled* scale gelatin, *Coregonus peled* scale gelatin phosphorylated by STMP for 0, 0.5, 1, 2 and 3 h respectively. For the same column, different superscripts indicate significant differences (*p* < 0.05).

**Table 8:** Secondary structure percentage (%) analysis of *Esox lucius* scale gelatin by analyzing the areas of 1600–1700 cm^−^^1^ in FTIR spectra.

Sample	β-Sheet	Random Coil	α-Helix	β-Turn	β-Antiparallel
ES	38.74 ± 3.39 ^b^	13.84 ± 0.79 ^a^	18.43 ± 8.57 ^a^	27.78 ± 4.79 ^a^	5.97 ± 1.49 ^a^
ES-0	42.12 ± 3.39 ^ab^	13.21 ± 0.66 ^a^	12.60 ± 0.36 ^a^	26.42 ± 4.91 ^a^	5.66 ± 0.50 ^a^
ES-0.5	42.28 ± 3.39 ^ab^	13.09 ± 0.61 ^a^	12.41 ± 0.39 ^a^	26.39 ± 4.88 ^a^	5.82 ± 0.50 ^a^
ES-1	43.31 ± 4.43 ^ab^	14.28 ± 1.53 ^a^	13.18 ± 1.39 ^a^	23.40 ± 4.09 ^a^	5.82 ± 0.90 ^a^
ES-2	45.44 ± 3.45 ^ab^	13.61 ± 0.57 ^a^	12.53 ± 0.25 ^a^	22.83 ± 4.74 ^a^	5.59 ± 0.46 ^a^
ES-3	46.84 ± 3.41 ^a^	13.44 ± 0.57 ^a^	12.37 ± 0.24 ^a^	21.98 ± 4.67 ^a^	5.37 ± 0.45 ^a^

ES, ES-0, ES-0.5, ES-1, ES-2, ES-3: *Esox lucius* scale gelatin, *Esox lucius* scale gelatin phosphorylated by STMP for 0, 0.5, 1, 2 and 3 h respectively. For the same column, different superscripts indicate significant differences (*p* < 0.05).

**Table 9:** Secondary structure percentage (%) analysis of *Grass carp* scale gelatin by analyzing the areas of 1600–1700 cm^−1^ in FTIR spectra.

Sample	β-Sheet	Random Coil	α-Helix	β-Turn	β-Antiparallel
GS	37.90±3.88 ^a^	13.33±0.72 ^a^	13.13±0.57 ^a^	29.07±5.56 ^a^	6.57±0.50 ^a^
GS-0	40.22±3.94 ^a^	13.13±0.66 ^a^	12.66±0.44 ^a^	27.49±5.22 ^a^	6.50±0.52 ^a^
GS-0.5	39.99±3.97 ^a^	13.41±0.59 ^a^	12.98±0.53 ^a^	27.59±5.34 ^a^	6.02±0.45 ^a^
GS-1	38.41±3.91 ^a^	13.29±0.72 ^a^	13.20±0.65 ^a^	28.90±5.50 ^a^	6.21±0.48 ^a^
GS-2	39.92±3.96 ^a^	13.75±0.60 ^a^	13.25±0.48 ^a^	27.21±5.41 ^a^	5.87±0.44 ^a^
GS-3	38.92±3.82 ^a^	13.26±0.73 ^a^	12.91±0.49 ^a^	28.33±5.37 ^a^	6.58±0.48 ^a^

GS, GS-0, GS-0.5, GS-1, GS-2, GS-3: *Grass carp* scale gelatin, *Grass carp* scale gelatin phosphorylated by STMP for 0, 0.5, 1, 2 and 3 h respectively. For the same column, different superscripts indicate significant differences (*p* < 0.05).

## Data Availability

The data presented in this study are available on request from the corresponding author.
